# Supplementary material for: Design, Synthesis, Bioactivity Evaluation, Crystal Structures, and In Silico Studies of New α-Amino Amide Derivatives as Potential Histone Deacetylase 6 Inhibitors
Source: Molecules. 2022 May 22;27(10):3335. doi: 10.3390/molecules27103335 (PMC9147695; doi:10.3390/molecules27103335)
Supplement: Supplementary file 1 [file molecules-27-03335-s001.zip › molecules-1653193-supplementary.pdf]

Supplementary informations

S1. Self docking

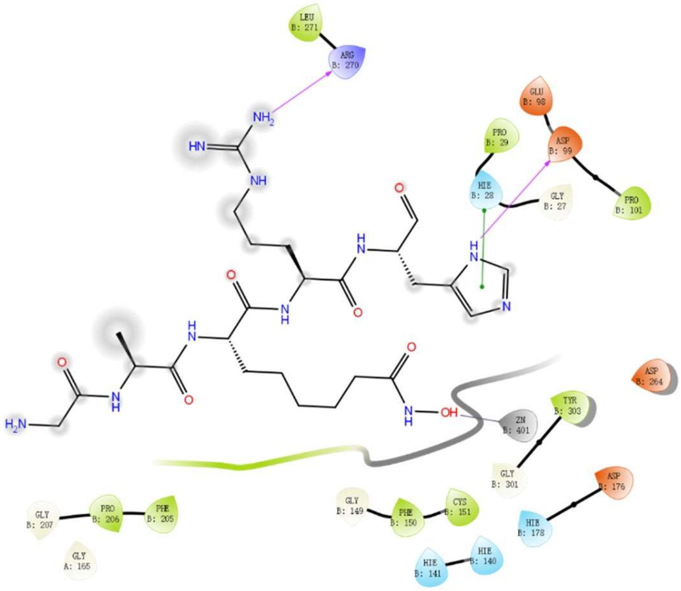

HDAC1： 5icn

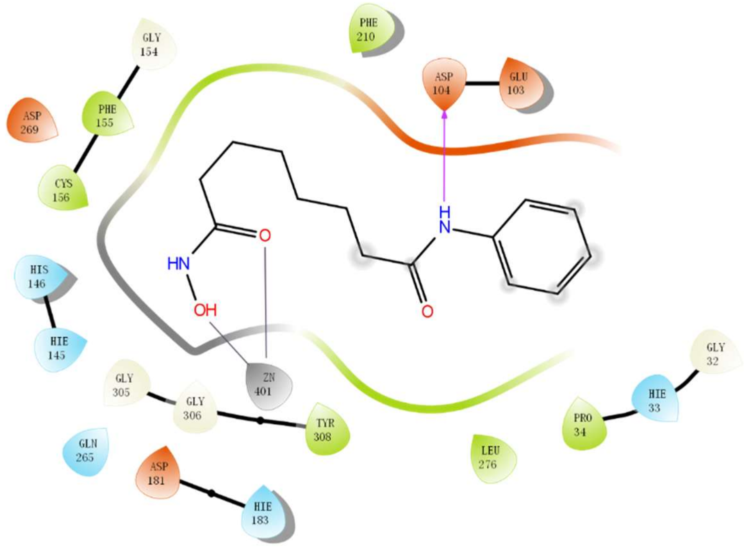

HDAC2: 4lxz

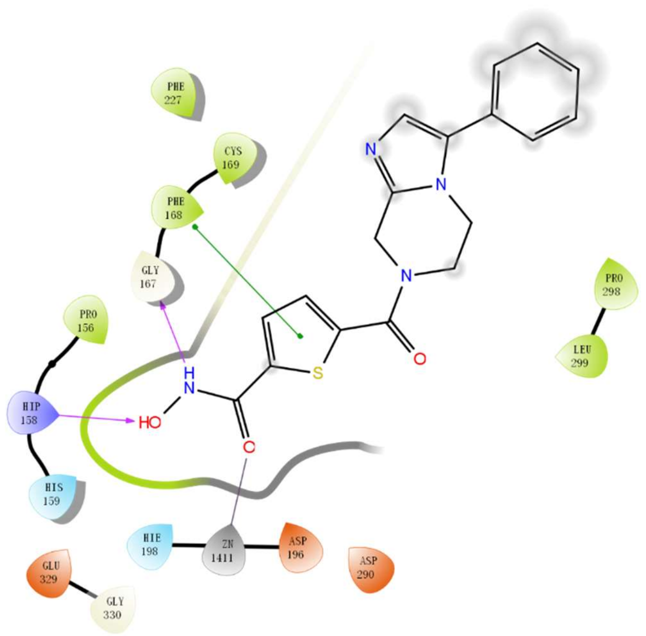

HDAC4 2vqm

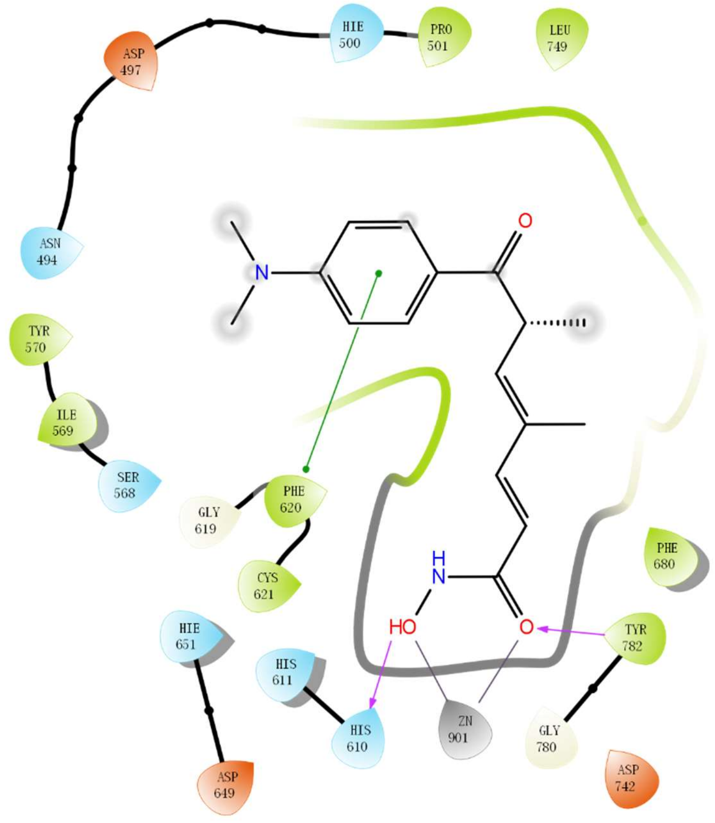

HDAC6:5edu

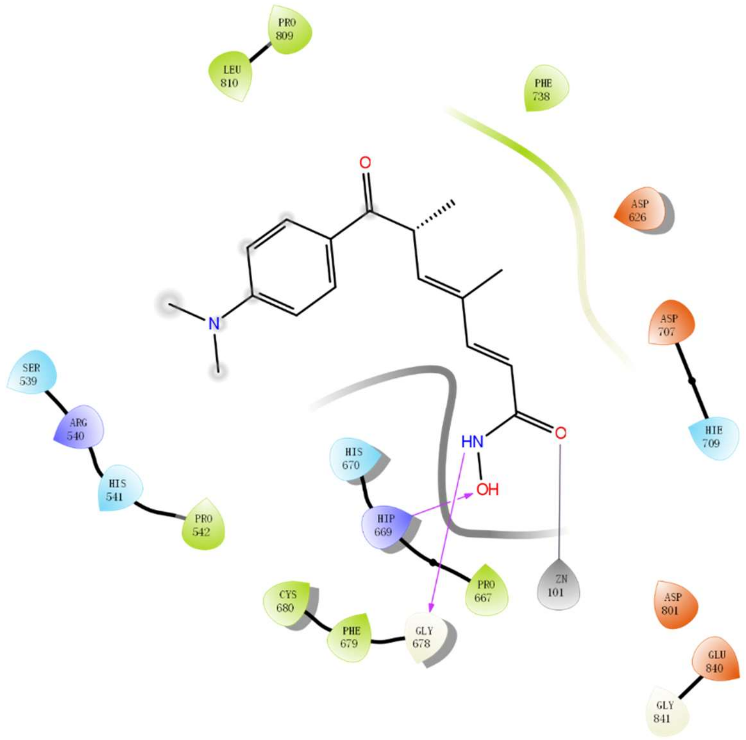

HDAC7:3c10

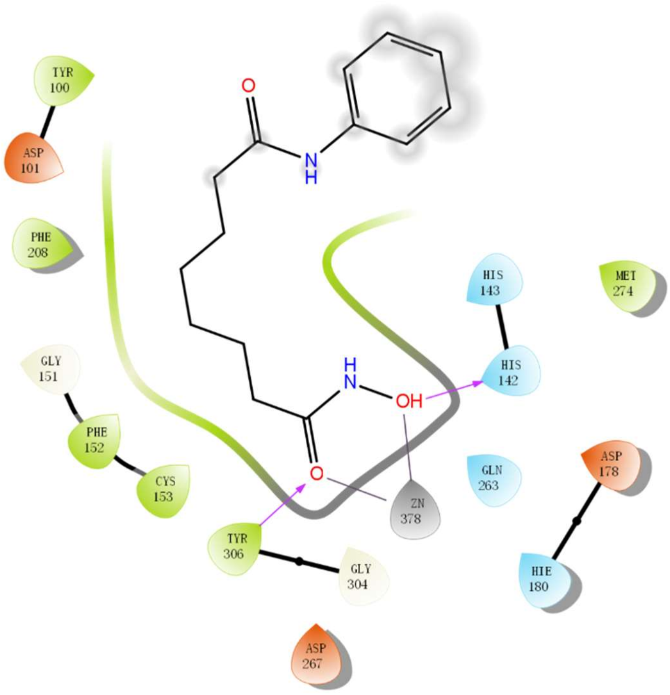

HDAC8:1t69

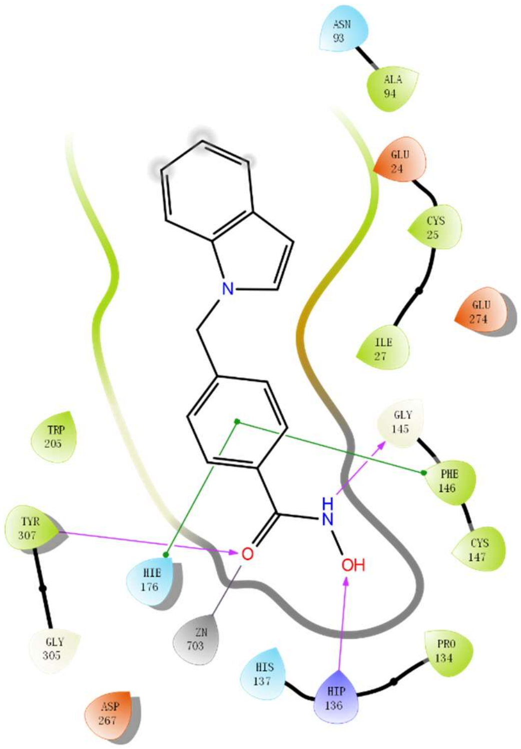

HDAC10：6wdy

S2. Reverse docking

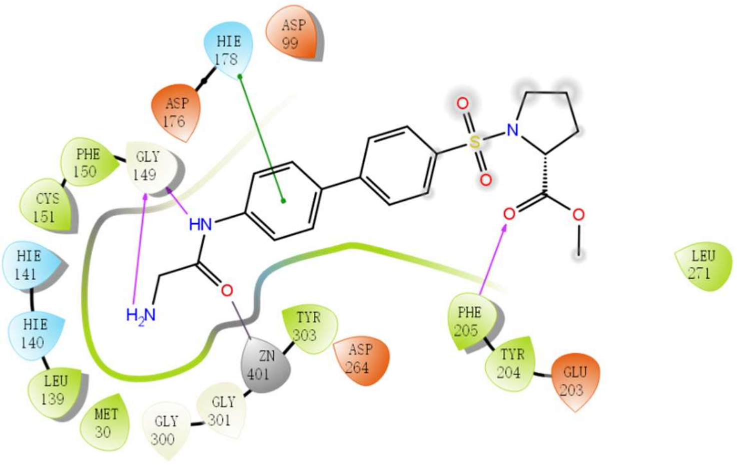

HDAC1

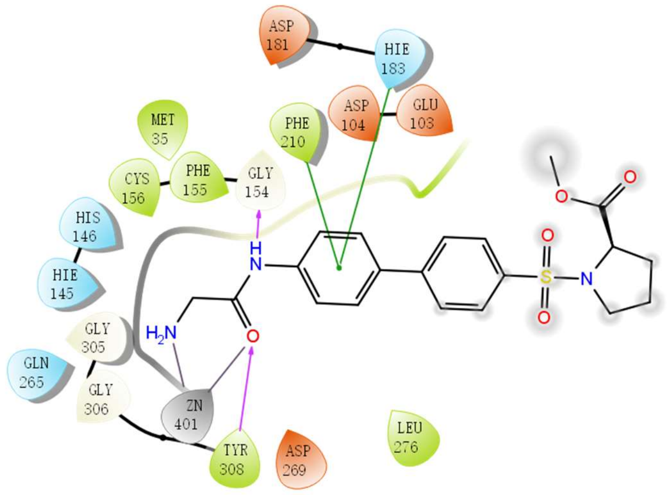
ASP 181
HIE 183
ASP 104
GLU 103
MET 35
PHE 210
CYS 156
PHE 155
GLY 154
HIS 146
HIE 145
GLY 305
GLN 265
GLY 306
ZN 401
TYR 308
ASP 269
LEU 276
H
N
H2N
O
O
S
O
N
O
O

HDAC2

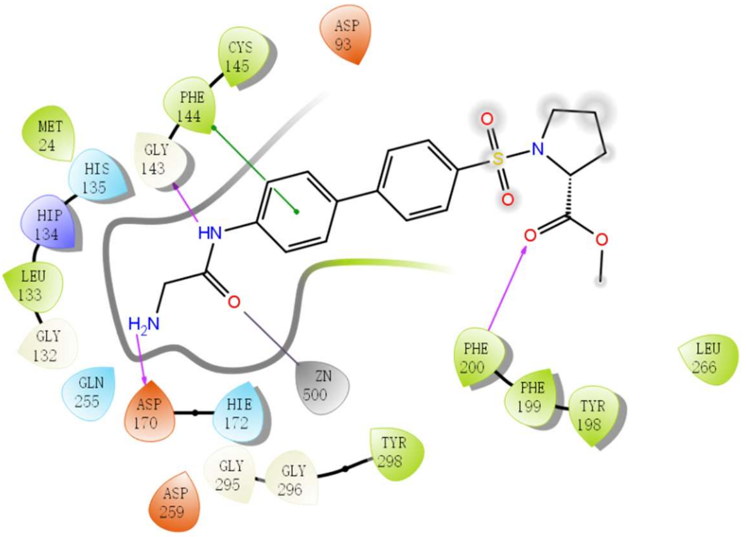
ASP 93
CYS 145
PHE 144
MET 24
GLY 143
HIS 135
HIP 134
LEU 133
GLY 132
GLN 255
ASP 170
HIE 172
ZN 500
PHE 200
PHE 199
TYR 198
LEU 266
TYR 298
GLY 295
GLY 296
ASP 259
HN
H2N
O
O
S
O
N
O
O

HDAC3

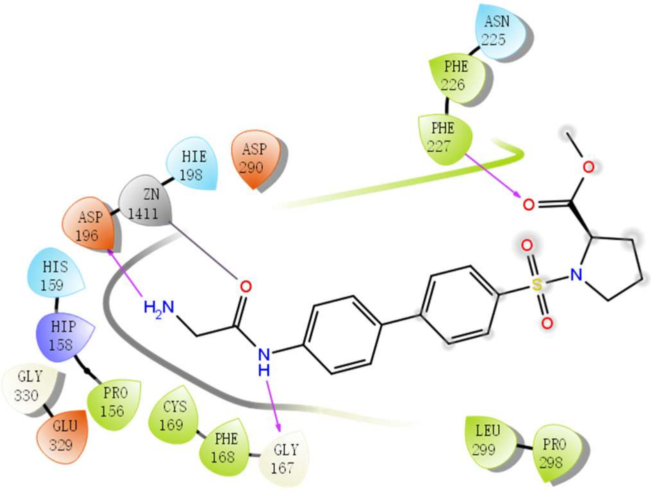
ASN 225
PHE 226
PHE 227
HIE 198
ASP 290
ZN 1411
ASP 196
HIS 159
HIP 158
GLY 330
PRO 156
GLU 329
CYS 169
PHE 168
GLY 167
LEU 299
PRO 298
H2N
O
N
H
O
S
O
N
O
O

HDAC4

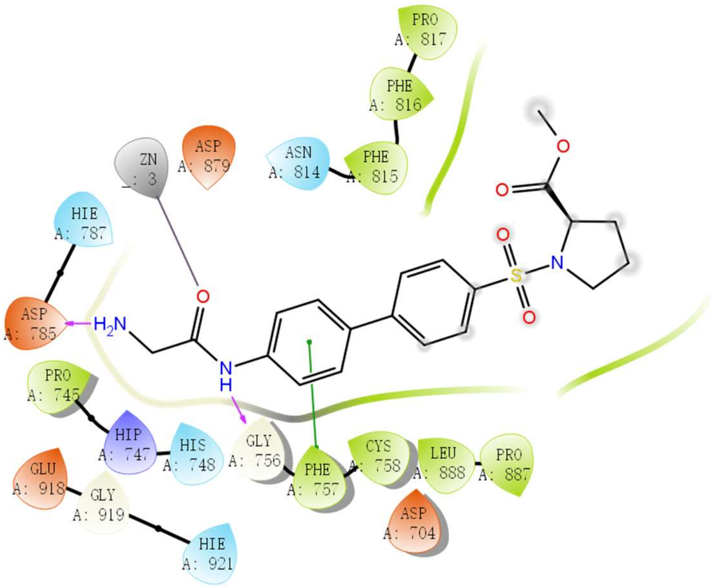

HDAC5

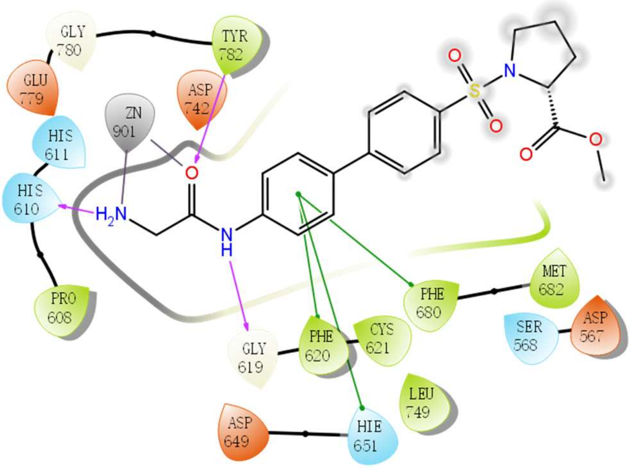

HDAC6

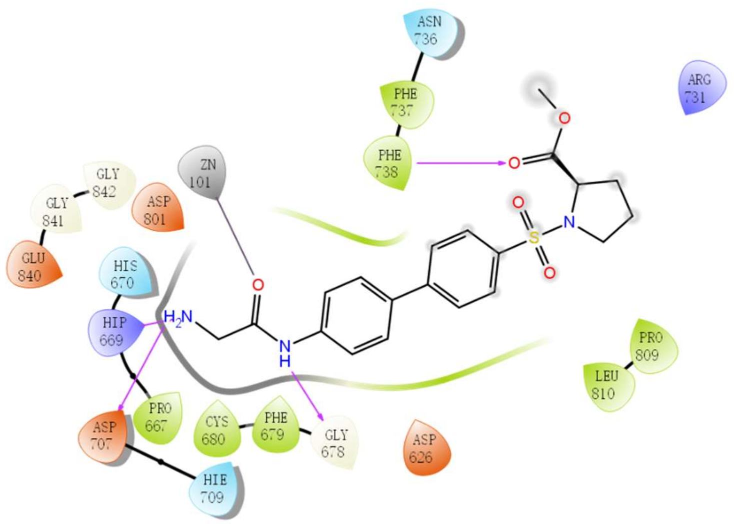

HDAC7

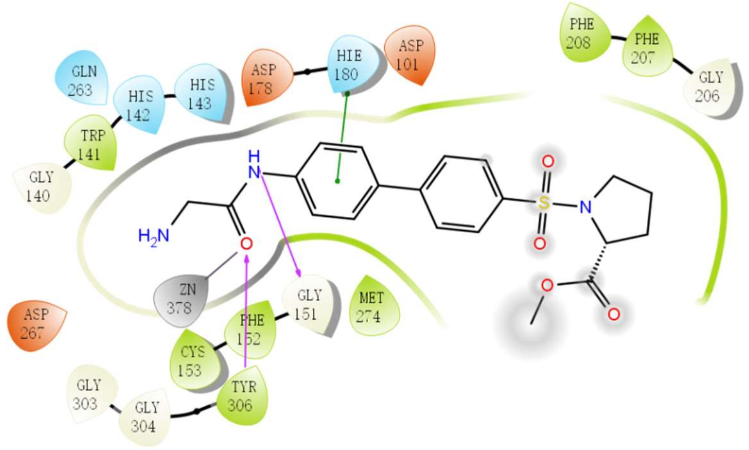

HDAC8

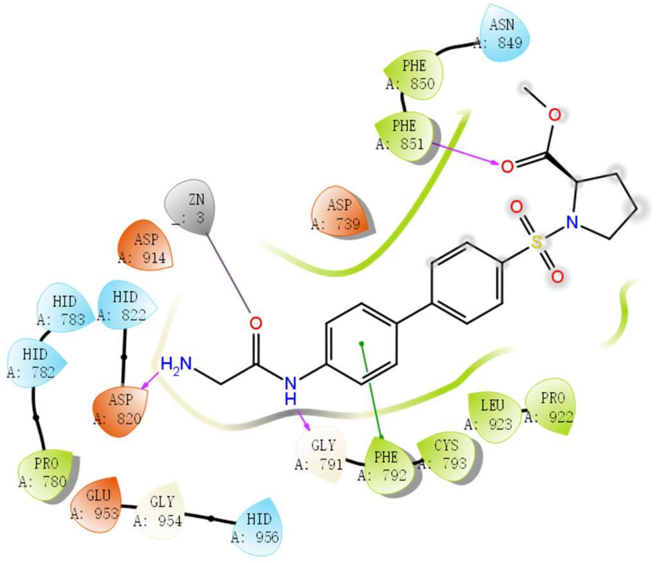

HDAC9

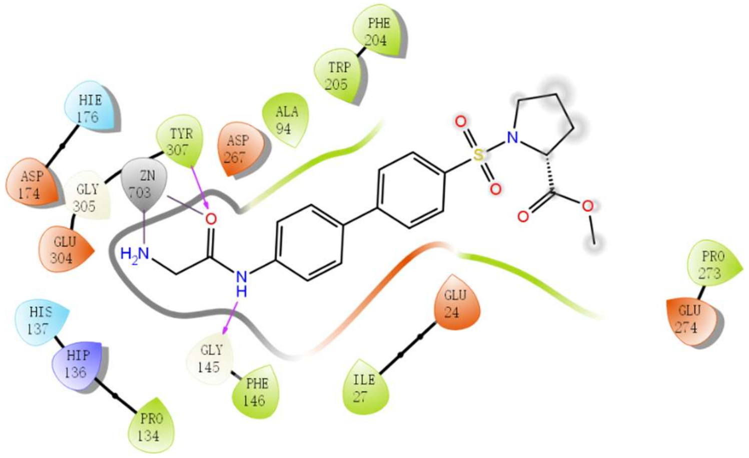

HDAC10
